# Maths anxiety and subjective perception of control, value and success expectancy in mathematics

**DOI:** 10.1098/rsos.231000

**Published:** 2023-11-29

**Authors:** Denes Szucs, Enrico Toffalini

**Affiliations:** ^1^ Department of Psychology, University of Cambridge, Cambridge CB2 1TN, UK; ^2^ Department of General Psychology, University of Padua, Padua, Italy

**Keywords:** maths anxiety (ma), expectancy-value theory, control-value theory, subjective factors, value of mathematics, motivation

## Abstract

Mathematics anxiety (MA) is an academic anxiety related to doing, learning and testing mathematics. MA can negatively affect mathematics performance, motivation and maths-heavy science and technology-related career choices. Previous data suggest that subjective perceptions and interpretations of students are key in the genesis of MA. Here, based on expectancy-value and control-value theory, we aimed to identify potential, theoretically based subjective factors probably key to understanding MA. We analysed data from 151 745 fifteen-year-old children from 65 ‘countries and economies' from the Programme for International Student Assessment (PISA) 2012 dataset. Subjective self-perceptions had a stronger relationship with MA than maths achievement. We found that higher MA was associated with lower perceived control over maths activities and lower subjective expectation of success. Surprisingly, children with higher subjective valuation of maths had higher MA for similar levels of subjective control and success expectancy in maths. Results offer an improved understanding of potential antecedents of MA and suggest that effective interventions could be based on gradual confidence building in maths. These could primarily draw on a deeper understanding of the subject improving subjective success expectancy and feeling of control over maths activities. Cultural variation in findings is discussed.

## Highlights

High MA is associated with low perceived control over maths and low subjective expectation of success in maths.

High MA is associated with relatively high valuation of maths.

Interactions and nonlinearities were minor in the data.

MA may be alleviated by gradual confidence building in maths.

There is notable cultural variation in subjective factors relevant to MA.

## 1. Introduction

Anxiety can be defined as a feeling of helplessness focused on future threats or threats to self-esteem [1]. Children are often anxious about academic domains in school. Moderate levels of anxiety can be performance boosting [2], but extreme levels of anxiety can have negative consequences: intrusive thoughts about worries and negative emotional states can limit attentional and working memory resources devoted to task performance [3–5] or can undermine long-term interest and intrinsic motivation: children may avoid certain academic subjects, may lose their motivation for doing them, and their quality of life may suffer [6]. Mathematics anxiety (MA), anxiety related to doing, learning and testing mathematics, is a particularly prevalent form of academic anxieties [7–9]. MA may be an important factor affecting student well-being and mental health, the liking of mathematics and whether students orient themselves toward maths-related career choices, typically science, technology, engineering and mathematics (STEM) careers, often associated with high earning potential [6,10,11]. Through the above mechanisms MA may also contribute to the shortage of STEM graduates in many countries. Considering the potentially significant impact of MA it is important to clarify the most important factors associated with it. Here, we aimed to identify potential antecedents of MA based on the expectancy-value theory (EVT) of achievement motivation and the control-value theory (CVT) of achievement emotions [12–15]. We used data from 151 745 fifteen-year-old children from 65 ‘countries and economies’ from the Programme for International Student Assessment (PISA) 2012 dataset. We tested how MA relates to perceived control over maths outcomes (Control), the subjective value attributed to maths (Value) and to Success Expectancy in maths. The results constitute a large-scale test of the predictions of EVT and CVT and bring us closer to understanding the context in which MA appears and develops.

MA is a specific anxiety related to maths testing and learning [16–19]. MA correlates negatively with maths achievement (*r* ≈ −0.3) in large samples and at the country level [20,21]. For example, in the PISA 2012 data, countries with the highest MA levels lagged by nearly a whole school year behind countries with the lowest levels of MA [21]. Contrary to frequent stereotypes, 80% of low maths achievers do not have high MA whereas 80% of students with high MA are normal to high achievers [20]. Hence, high MA affects a very large proportion of children. MA is consistently higher in females than in males [21] even in samples where females perform at the same level as males [18,20,22]. Academic anxieties and MA probably become more specifically linked to school performance as children progress through the school system [23]. Such development of MA could in principle be based on specific experiences in school. However, a large UK study that collected both quantitative and interview data could not identify any unique triggers of MA and concluded that subjective factors, such as the interpretation of events, may be key to understanding the origins of MA [24].

Influential theories of achievement motivation and emotions, EVT [12–14] and CVT [25], identify subjective factors that may be key to understanding MA. Achievement can be defined as ‘the quality of activities or their outcomes as evaluated by some standards of excellence’ [26, p. 15]. EVT focuses on identifying the determinants of *achievement motivation*, educational engagement with a subject and wider educational choices. EVT identifies *Success Expectancy* and *subjective task value* as prime determinants of achievement motivation and subject engagement: Success Expectancy is probably strongly linked to academic achievement while task value would primarily correlate with academic career choices [12–14].

While EVT focuses on achievement motivation, CVT aims to explain how achievement emotions [25], domain-specific [27] emotional states associated with achievements, are generated. Achievement emotions are undoubtedly important as they can strongly influence students' motivation to engage with certain subjects [11,28,29]. CVT suggests that achievement emotions are, on the one hand, determined by the perceived subjective control level (low/high) over achievement outcomes (e.g. completing a test) and achievement activities (e.g. doing maths in general) as well as by the attribution of control (internal/external). On the other hand, CVT suggests that value assessments form another major factor shaping achievement emotions. These are the subjective value of achievement outcomes (positive/success versus negative/failure expectations) and activities (e.g. the positive/negative intrinsic value of studying maths irrespective of grades or the extrinsic/utility value of maths due to parents’ valuation) [25,26,30]. Achievement emotions are classed as pleasant or unpleasant (e.g. joy versus anxiety), activating or deactivating (e.g. enjoyment versus boredom), focused on prospective or retrospective outcomes (e.g. future or past maths tests) or activities rather than outcomes (e.g. doing maths irrespective of testing).

CVT conceptualizes anxiety as a *prospective outcome emotion* that appears when the prospective (future) outcome value of an achievement-related event is negative (i.e. Success Expectancy related to sitting a maths test is negative/low) and the perception of control is medium ([25], pp. 320–321). By contrast, when students' prospective outcome value is positive (i.e. they expect to sit a maths test successfully), and they feel that they have high control over the outcome, then CVT expects that ’anticipatory joy’ will dominate as achievement emotion: students will be confidently looking forward to their successful test completion. CVT assumes that higher subjective task/activity value results in stronger emotions tied to a task [26]. For example, the higher the valuation of a task/activity, the higher the anxiety linked to the task/activity.

Importantly, the above conceptualization of anxiety by CVT and the emphasis on subjective factors by both EVT and CVT suggests that high MA is not simply the consequence of inadequate maths subject knowledge in school (for review see Carey *et al*. [31]). First, it is the subjective perception of control over maths that matters rather than ’objective’ achievement levels. Hence, even some (relatively) high achievers may perceive their achievement level as inadequate (e.g. depending on their peer comparison group or on parental expectations). Second, high MA would appear in students who value maths relatively highly but not in the ones who attribute relatively low value to maths. These assumptions fit well with research data reporting that about 80% of low maths achievers do not have high MA whereas about 80% of children with high MA are normal to high achievers [20]. Based on EVT and CVT it could be argued that many objectively low achievers may not value maths high enough to develop high MA. By contrast, many objectively good/high achievers may value maths high enough and may consider their achievement level inadequate enough to develop high MA [32].

### 1.1. Past relevant empirical research

Relatively few studies relied on EVT and CVT in examining MA as an achievement emotion and students' related educational choices. These studies used various questionnaires with subscales not necessarily labelled consistently across studies: often, questionnaires with very similar items were measuring concepts labelled differently. First, based on EVT, some studies measured *Value, the subjective task value of maths* by asking about the intrinsic, utility and achievement/attainment value of maths as well as parental valuations of maths [33–35].

Second, studies also measured concepts similar to that of *‘prospective outcome value’* of CVT and *Success Expectancy* in EVT [13,25]. Some studies asked about Success Expectancy in concrete situations, classing questionnaires as measures of *’success expectancy’* or *’self-efficacy’*. Such task-specific questions are most typically considered [13] measures of ’self-efficacy’ [36], referring to a ‘conviction that one can successfully execute the behaviour required to produce the outcomes' [37, p. 193], [38]. Classical theory assumes that self-efficacy beliefs determine goal-setting, actions taken and the invested effort and perseverance of action [38].

Further, some studies asked more general questions about Success Expectancy. Instruments were referred to as measures of ‘*maths ability self-concept’* or *’perceived maths ability*’ [39]. While the previously mentioned self-efficacy measures are thought to primarily rely on cognitive appraisals about students' abilities, self-concept measures are thought to be more complex, including emotional and social comparison elements [40]. That is, self-efficacy measures are more focused on certain tasks, while self-concept measures are more global: self-efficacy perceptions may serve as precursors of self-concept [41]. While self-efficacy and self-concept measures refer to different constructs, they both undoubtedly involve a focus on perceived competence and expected success. So, both types of measures can be thought to measure Success Expectancy or ’prospective outcome value’. Consequently, it has been argued that maths ability self-concept questionnaires measure the same construct as maths Success Expectancy measures [33].

Third, a few studies based on CVT used declared measures of *Control* [34,42,43]. It has been argued that maths-related low/high control perceptions can be distinguished from self-efficacy beliefs because control perceptions focus on ’performance of an activity (e.g. investing effort during learning) rather than on success at solving a problem’ [42, p. 1343]. Nevertheless, control and self-efficacy measures probably have some interdependency: high self-efficacy and self-concept perceptions are probably related to experiencing strong control over a behaviour and attributing such control to the self (unless one believes that one has endless luck in executing appropriate behaviours during maths tests).

Considering results, when a Control measure was conceptualized, negative correlations and path coefficients were found between this measure and MA [34,43]. Regarding Success Expectancy-related measures, all studies that used these (measures named as ’success expectancy’, ’perceived maths ability’, ’maths self-efficacy’, ’maths (ability) self-concept’) reported negative correlations and/or negative SEM path coefficients between the measures and MA [10,33–35,43–48].

Empirical results about measures of Value are mixed. Some studies reported negative zero-order correlations but positive path coefficients between measures of utility value and intrinsic value and MA [10,46,47]. Lauermann *et al*. [35] reported positive correlations and path coefficients between intrinsic and utility value and parental valuing of maths and a single questionnaire item on worry about mathematics and reading. The above results were interpreted to suggest that MA may appear when children value mathematics highly and attribute high importance to performing well in maths but at the same time they have low success expectancy, or they rate their ability low in the subject [10,32,35]. Such attitudes could also explain why girls often have higher MA than boys [32] even if their MA is not justified by their performance [21,49].

In contrast to the above, other studies reported negative structural equation modelling (SEM) path coefficients and zero-order correlations between Value and MA [34,44]. Putwain & Wood [34] measured intrinsic, attainment (e.g. ‘I want to get good marks in maths') and utility value merged into a single scale and concluded that results may suggest that students who do not expect to be successful and have high MA may devalue maths in order to protect their self-worth (i.e. high MA would lead to low Value). This causal process would result in an ultimately negative correlation between Value and MA [34]. As described above, most studies measured the intrinsic and utility value of maths and one study considered parental valuations affecting the value of maths Lauermann *et al*. [35]. The PISA 2012 data also included questions not only about the intrinsic and utility value of maths but also about parental and peer valuations of maths.

In summary, the literature suggests that Control and Success Expectancy are negatively related to MA (higher control and higher success expectancy are associated with lower MA) whereas it is not clear whether higher or lower Value is linked to higher or lower MA.

### 1.2. The current study

In this theory-driven study we determined whether the predictions of EVT and CVT regarding MA hold in the PISA 2012 dataset of 151 745 fifteen-year-old children from 65 ‘countries and economies’ [21]. We have used all the variables critical to EVT and CVT in a single framework: we considered the subjective task value of maths, Control over maths as well as Success Expectancy in maths. We considered not only the intrinsic and utility value of maths but also the valuations of parents and friends. First, we aimed to replicate previous results from smaller studies that found that higher Control and Success Expectancy were related to lower MA. Second, we aimed to resolve inconsistencies in previous results by determining whether higher or lower Value was associated with higher or lower levels of MA. Third, while CVT originally assumed that anxiety was associated with medium level of Control [25], empirical studies suggest that low levels of Control are associated with the highest level of MA [34,43]. Here, we have contrasted these options. Fourth, interactions between variables have rarely been explored in detail ([35,43,50], sample sizes = 120, 1298, 805). Here, we investigated potentially complicated multi-variable interactions. The very large sample used here was particularly adequate to this purpose. Fifth, the international coverage of the data enabled us to explore cultural variation across the 65 observation units represented in the PISA dataset. Understanding cultural variability is particularly important as the reporting and conceptualization of anxiety and related subjective factors are probably subject to substantial cultural influence.

## 2. Methods

### 2.1. Data extraction and preprocessing

PISA 2012 [21] data was accessed at https://www.oecd.org/pisa/data/pisa2012database-downloadabledata.htm using the access provided by the University of Cambridge. The data was downloaded in text format and extracted by a custom Matlab (www.mathworks.com) script. In our analyses we used PISA variables CNT (country code; hereafter ’Country’) and gender (ST04Q1) as well as questionnaire data extracted from PISA variables ST29Q01 to ST44Q08 (see pages 1, 64 and 95–109 of the PISA codebook ‘PISA12 stu codebook.pdf’). Gender was coded as 1 = Female; 2 = Male. We have analysed data from the following PISA questionnaire variables: Maths interest; Instrumental motivation; Subjective norms; Perceived control; Attributions to Failure; Maths self-efficacy, Maths self-concept; Maths Anxiety (see the above codebook pages for details and questions). The variable ’Work Ethic’ was also extracted but not used.

Students could respond to most questionnaire items with four valid response options: ‘Strongly agree = 1’, ‘Agree = 2’, ‘Disagree = 3’ and ‘Strongly disagree = 4’, except in case of maths self-efficacy where options ranged from ‘Very confident = 1’ to ‘Not at all confident = 4’. The above response options were mapped to numerical values 1 to 4 in PISA data files. Other response codes in PISA data represented N/A (7), invalid (8) or missing (9) data. After extracting PISA data, the frequencies of extracted response values for each PISA variable were matched against the frequencies reported in the PISA codebook (see above) to ascertain data fidelity.

During preprocessing, responses to individual questionnaire items were recoded so that higher valid scores (maximum = 4) represented higher value on measured constructs (e.g. more anxiety, stronger perception of control, etc.) than lower valid scores (minimum = 1). Invalid scores (7–9) were replaced with NaN (not a number) values. Questionnaire item scores were then averaged to create an individual questionnaire score for each child. If any of the questionnaire items were NaN, then the relevant questionnaire average was also NaN due to the NaN item score. Children with invalid NaN questionnaire average scores in any of the questionnaires were removed from analysis. That is, only children with no missing or invalid items in any of the questionnaires were kept for analysis. The initial datafile contained data from 480 174 children. After removing children with missing or invalid data, the database had data from 151 745 children, from 77 252 girls and 74 493 boys from 65 observation points. Children in the sample had birth dates of 1996 and 1997. The recoded and cleaned data was saved into a text file. The data was analysed further in R and Python.

### 2.2. Constructs

We have computed scores for the below constructs defined by the ‘PISA 2012 Results: Ready to Learn’ booklet [21]. (1) ‘Perceived control’, defined by questions based on ‘student responses about whether they strongly agree that success in mathematics and school depends on whether they put in enough effort’ (p. 65 of the OECD booklet). (2) ‘Attributions to failure’, defined by questions ‘based on students’ responses about whether they attribute failure in mathematics tests to themselves or to others' (p. 65). (3) ‘Maths interest’ (Intrinsic motivation) and (4) ‘Instrumental motivation’ [hereafter ‘Utility’], defined by questions ‘based on students’ responses about whether they enjoy mathematics and work hard in mathematics because they enjoy the subject, and whether they believe mathematics is important for their future studies and careers' (p. 65). (5) ‘Subjective norms’, defined by questions ‘whether students’ parents and peers enjoy and value mathematics' (p. 88). At data extraction we distinguished between the two reference groups used by the PISA questions as three questions asked about parents’ and three questions asked about friends' valuation of mathematics. So, we created ‘Parents’ and ‘Friends’ scales from the relevant questions for an initial modelling attempt described below. (6) ‘Maths self-efficacy’, defined by questions ‘based on students’ responses about their perceived ability to solve a range of pure and applied mathematics problems' (p. 88). (7) ‘Maths self-concept’, defined by questions ‘based on students’ responses about their perceived competence in mathematics' (p. 88). (8) ‘Maths anxiety’, MA, defined by questions ‘based on students’ responses about feelings of stress and helplessness when dealing with mathematics' (p. 88). Questionnaire items assigned to constructs can be seen in the above-cited PISA codebook.

Here, we used the above measures to characterize the key constructs of EVT and CVT: Control (over maths), Value (of maths) and Success Expectancy (regarding maths). Control was measured by ‘Perceived control’ and ‘Attributions to failure’. ‘Attributions to failure’ was included in our model because (as described in the Introduction) CVT assumes that the attribution of control is an important element of Control perceptions. Success Expectancy was measured by ‘Maths self-efficacy’ and ‘Maths self-concept’. Value was measured by ‘Maths interest’ (Intrinsic motivation), ‘Instrumental motivation’ (referred to below as Utility value to be in line with the relevant literature) and ‘Subjective norms' (including parents' and friends’ perceived valuation of mathematics). In an initial modelling attempt we further distinguished between self-related value (Value-self: ‘Maths interest’ and ‘Instrumental motivation’) and socially grounded values (Value-social: ‘Subjective norms’) [35]. Such a distinction between individual and social determinants of valuations could be justified in principle [35].

### 2.3. Data analysis

All data analysis and visualization was performed in R, v. 4.3.1 [51], and Python, v. 3.9.7 [52] using the following packages: ‘lavaan’, ‘lme4’, ‘splines’ (R) for model fitting, and ‘ggplot2’ (R) and ‘matplotlib’ (Python) for data and effects visualization. Most data analysis was conducted under the framework of multi-level confirmatory factor analysis (CFA) and linear mixed-effects models (LMM). This allowed us to account for the variability of the effects across countries. ‘Country’ was always entered as a cluster in multi-level CFA and as a random effect in LMM with both random intercepts and random slopes. In LMM, the mean country-level scores of predictors were added as predictors in the formula, for all predictor variables which presented variability across countries (that is, all of them). This was done to avoid violating the Gauss–Markov assumption of exogeneity as well as to estimate possible level-2 (between-country) effects [53] obviously, no random slopes were estimated for these effects in LMM, as they represent between-country differences. In LMM, MA was the dependent variable of interest to model. Possible nonlinearities were examined with natural splines (only at the level-1 individual level). Statistical inference on model coefficients was made with reference to the *p*-values (*α* = 0.01), but given the large sample size, more emphasis was put on the consideration of estimated effect sizes. Considerations on the effects and their relevance were mostly made via visual inspection of plotted effects with their uncertainty bounds (95% confidence intervals (CIs)). In all statistical models (both CFA and LMM) observations were weighted for students' weights as provided by the PISA database. This is done because PISA oversample some target subpopulations within countries, leading to potentially biased results when observations are not weighted [54].

All effects of interest on MA were controlled for Maths Achievement. The latter was entered as a predictor of MA in all models. Maths Achievement was estimated using the first of the five alternative ‘plausible values’ provided by PISA. We choose to pick only one of the alternative values, instead of using all and averaging model estimates, for practical reasons. First, the PISA manual for statistical analysis [54] states that population estimates are unbiased for any of the five alternative values of Maths Achievement. Second, using alternative plausible values might lead to more accurate standard errors of estimates, but according to the manual this becomes practically negligible when more than a few thousand observations are analysed. Finally, recomputing and averaging was problematic due to the long computational times and the complexity of some fitted models, featuring interactions, random effects and even smoothers for nonlinear relationships.

In a first phase, we tested the goodness of fit of a multi-level measurement model for the predictors of interest (except Maths Achievement, which was directly represented by its plausible value estimated by PISA itself). Latent variables were composed using the subtotals computed from the PISA data. In the first multi-level CFA model, fitted latent values were Value-social (Friends + Parents), Value-self (Utility + Interest), Success expectancy (Self-efficacy + Self-concept), and Control (Control + Failure Attributions). In a second model, Value-social and Value-self were combined in a single Value latent variable. As we were interested in modelling variables mainly at the between-individual level, level-2 (between-country) was used only to control for differences in mean levels by entering random intercepts for observed variables across countries. Goodness of fit was assessed using the following standardized fit indices: root mean square error of approximation (RMSEA *<* 0*.*05 indicates good fit); comparative fit index (CFI *>* 0*.*95 indicates good fit); non-normed fit index (NNFI > 0*.*95 indicates good fit).

Once the measurement model was established, factor scores were calculated by averaging the observed variables. This was preferred over extracting the predicted values from multi-level CFA because this would lose between-country variability as random intercepts were entered for countries. All variables (including MA) were then rescaled to z-scores according to a standard normal distribution. For ease of interpretation, these z-scores were set to have s.d. = 1 after removing between-country mean differences. To do so, variables of interest (MA, Value, Success Expectancy, Control, Maths Achievement) were entered as dependent variables into LMMs, with only a fixed intercept and a random intercept for country. Each variable was then rescaled to have mean = 0 and was divided by the s.d. of the LMM residuals (i.e. s.d. of overall within-country variability). Finally, all predictor variables were split into a within-country component (mean-centred by country average), and a between-country component (i.e. the estimated country average) for ease of interpretation in the subsequent analyses. Country average values were estimated as the country random intercepts of the LMMs described above. As we used these z-scores, we interpreted model parameters as standardized coefficients with respect to within-country variability of MA.

In the main analysis, we modelled MA via LMM. We examined the linear main effects of each of its potential predictors (that include all the above plus Gender). Risk of collinearity in LMM was determined by investigating the variance inflation factors (VIFs) of the predictors, which indicates the ratio of increase of the estimated variance of model coefficients when covariates are entered in the model vis-a-vis when they are not entered. The typical rule-of-thumb cut-offs for VIF are 5.0 or 10.0: higher values indicate strongly multi-collinear predictor variables. Subsequently, we examined nonlinearities in all relationships under investigation by using spline regressions. This is a non-parametric technique that provides nonlinear fit by means of piecewise polynomial functions fitted across pre-specified intervals of data, defined by the number of ‘knots’, or discontinuities on the x-axis (predictor) continuum. We added natural cubic splines for all predictors of interest in the LMM using the ‘splines’ package of R, in the fixed part of the LMM. To avoid overfitting, only four knots were set for spline regressions. Nonlinearities emerging with more knots would also be difficult to interpret. Interpretation was conducted mainly through visual inspection of the fitted curves.

The final part of the analysis was exploratory. We examined up to two-way interactions among predictors on MA. An interaction implies that the effect of one predictor is different for different level of another predictor. For simplicity, we did not test higher-order interactions, and fitted all effects as linear. Also, interactions were examined only at level-1 (i.e. between-individual level), as the level-2 (countries) did not present enough observations (coded countries were only 62 as Mainland China had three observation points: Shanghai, Hong-Kong and Macao; Russia had two observation points: One general and Perm). Finally, we examined the random effects by visually inspecting the heterogeneity of the main effects across countries on a world map.

## 3. Results

### 3.1. Model

**Figure 1.**
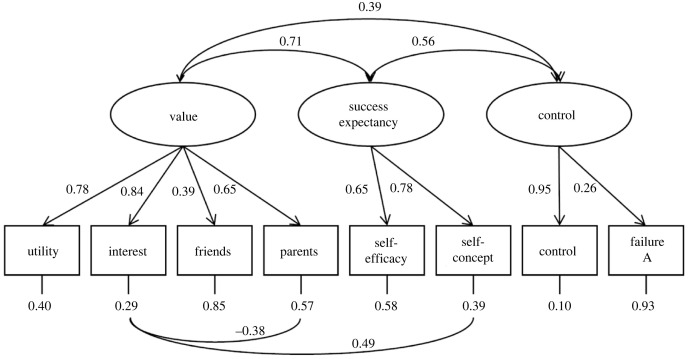
Summary of CFA model. Standardized coefficients are presented for level-1 (between individual). Random intercepts were entered for countries at level-2. In the bottom row of the figure ‘control’ refers to ‘Perceived Control’ and ‘failure A’ refers to ’Failure attributions'.

The first multi-level CFA model (fitting latent variables for Value-social, Value-self, Success expectancy, Control; see above), had very good fit only if residuals were correlated between Maths Interest and both Self-concept and Parents, RMSEA = 0.04, CFI = 0.98, NNFI = 0.97. However, inspection suggested that the latent variables Value-social and Value-self were correlated *r* > 0.99, proving to be entirely redundant. Also, the covariance matrix of latent variables was not positive definite. The second multi-level CFA model, shown in figure 1, featured a single Value factor, also had a good fit, RMSEA = 0.04, CFI = 0.97, NNFI = 0.96, and it did not present redundant factors. Hence, this model was considered technically acceptable. However, for subsequent analyses we only used the ‘Perceived Control’ score to represent the Control factor because ‘Failure attribution’ had a very small loading and it was also conceptually less relevant.

### 3.2. Correlations

Table 1 shows the correlation table of all variables of interest. Correlations were computed on country-centred z-scores to remove covariance due to between-country differences (unlike in the other model-based analyses, individual observations were not weighted here, for simplicity). The table also reports the estimated heterogeneity of z-scores of each variable between countries (*τ*) and the estimated percentage of total variance explained by heterogeneity (I2). Due to the *z*-score calculation described above, the variability of each country-centred variable (*σ*) was set to exactly 1.00. As shown, Value and Success Expectancy had medium-strong negative correlations with MA, while Value had weak negative correlation with MA. Value, Success Expectancy and Control were all moderately to strongly positively associated with each other.

**Table 1.** Correlations between country-centred z-scores, the estimated heterogeneity of z-scores of each variable between countries (*τ*) and the estimated percentage of total variance explained by heterogeneity (I2). Due to the z-scores calculation, the estimated residual variability (*σ*) is exactly 1.00 for each variable.

z-scores	1.	2.	3.	4.	5.
1. maths anxiety	—				
2. value	−0.16	—			
3. success expectancy	−0.47	0.55	—		
4. control	−0.40	0.32	0.43	—	
5. maths achievement	−0.34	0.07	0.44	0.30	—
estimated s.d. across countries (*τ*)	0.26	0.44	0.19	0.18	0.61
*I^2^*	7%	18%	4%	3%	32%

**Figure 2.**
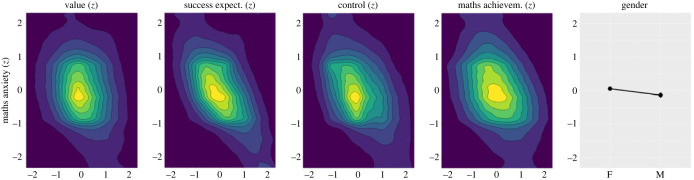
Two-dimensional density plots of MA (vertical axis) and its potential predictors (horizontal axis). The rightmost panel shows mean gender difference in Maths Anxiety. Colour represents density: darkest = (0.00, 0.02], lightest = (0.20, 0.22].

Figure 2 shows two-dimensional density plots featuring MA on the vertical axis and each of its potential predictors on the horizontal axis, along with the mean gender difference in Maths Anxiety. This figure illustrates the correlations represented in table 1 and depicts the weak impact of Gender on MA.

### 3.3. Linear main effects on maths anxiety

As described in the Data analysis section, a LMM was fitted with MA as the dependent variable. The summary is reported in table 2. The model converged correctly with 20 000 iterations with the ‘bobyqa’ optimizer. The largest VIF was 2.02, with the maximum increase in standard error amounting to 42% (for Success expectancy within-country). All fixed within-country effects were significant with *p* < 0.001, while only the between-country effect of Maths achievement was significant. All effects, except that of Success expectancy, were small to medium in terms of the effect size (note that, since all variables were standardized, regression coefficients can be directly interpreted as standardized effect sizes). Concerning the fixed effects, Value had a small positive association with MA, while the estimated effects of Success expectancy and Control were both negative. The net effect of Gender was negative, albeit with a negligible effect size, suggesting lower MA in males than in females even after controlling for all other predictors. Maths achievement had a small effect size at the within-country level, and a slightly larger effect size at the between-country level; both effects were negative and significant. Overall, fixed effects in the model explained a combined 30% MA variance, while adding the random effects led to 38% of MA variance explained by the model. The estimated s.d. of the random slope for country suggested moderate but not irrelevant heterogeneity of all effects across countries, which will be examined in the last subsection of the Results.

**Table 2.** Summary of the LMM on MA as the dependent variable. *N* = 151 745 observations. *Note*. VIF = variance inflation factor.

response: maths anxiety	fixed effects			VIF	random effects
	estimate	95% CI	*p*-value		*τ* (s.d.) country
*within-country*					
(intercept)	−0.01	(−0.07, 0.04)	0.600	—	0.21
value	0.14	(0.13, 0.16)	<0.001	1.34	0.06
success exp.	−0.42	(−0.46, −0.39)	<0.001	2.02	0.15
control	−0.24	(−0.26, −0.23)	<0.001	1.06	0.06
maths achiev.	−0.08	(−0.10, −0.06)	<0.001	1.91	0.08
gender	−0.05	(−0.07, −0.03)	<0.001	1.18	0.07
*between-country*					
value	−0.03	(−0.14, 0.08)	0.591	1.68	
success exp.	0.10	(−0.10, 0.13)	0.330	1.08	
control	0.19	(−0.04, 0.41)	0.099	1.07	
maths achiev.	−0.17	(−0.25, −0.09)	<0.001	1.71	
marginal *R*^2^	0.30				
conditional *R*^2^	0.38				

**Figure 3.**
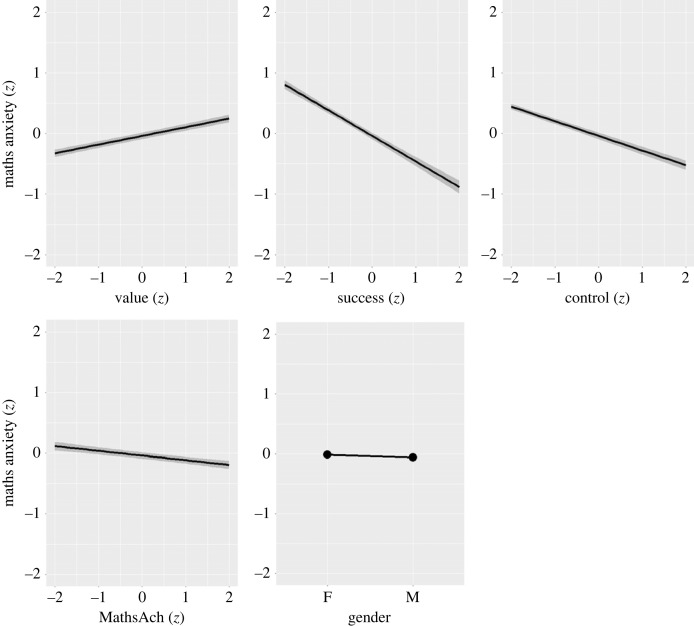
Estimated (linear) fixed effects on maths anxiety. Shaded areas represent 95% confidence bands.

Figure 3 shows the estimated linear fixed effects of all predictors on MA, excluding the between-country effects, which were estimated as practically zero.

### 3.4. Check of possible nonlinearities

We have exampled possible nonlinearities in the data and concluded that these were negligible. Details are shown in electronic supplementary material, figure S1.

### 3.5. Interactions on maths anxiety

**Figure 4.**
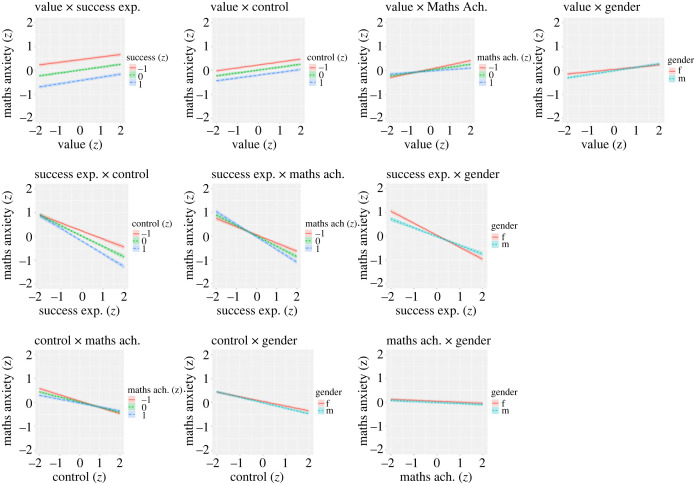
Plot of two-way interaction effects. All terms are treated as linear.

An additional LMM was fitted, in which all two-way interactions were entered. The model presented convergence issues, which were resolved by not modelling the random intercept-slope covariance (the coefficients reported below remained virtually identical in any case). Most interaction coefficients were statistically significant, but always small or even negligible in magnitude, in the fixed part of the model (Value × Success: B = −0.01, *p* = 0.01; Value × Control: B = −0.002, *p* = 0.58; Value × Maths Ach.: B = −0.06, *p* < 0.001; Value × Gender: B = 0.05, *p* < 0.001; Success × Control: B = −0.10, *p* < 0.001; Success × Maths Ach.: B = −0.09, *p* < 0.001; Success × Gender: B = 0.14, *p* < 0.001; Control × Maths Ach.: B = 0.05, *p* < 0.001; Control × Gender: B = −0.03, *p* < 0.001). Interactions are more easily interpreted via visual inspection; figure 4 reports predicted effects for all of them. In virtually all cases, the difference in slopes across different levels of the moderator variable are very small to virtually invisible.

### 3.6. Heterogeneity of effects across countries

**Figure 5.**
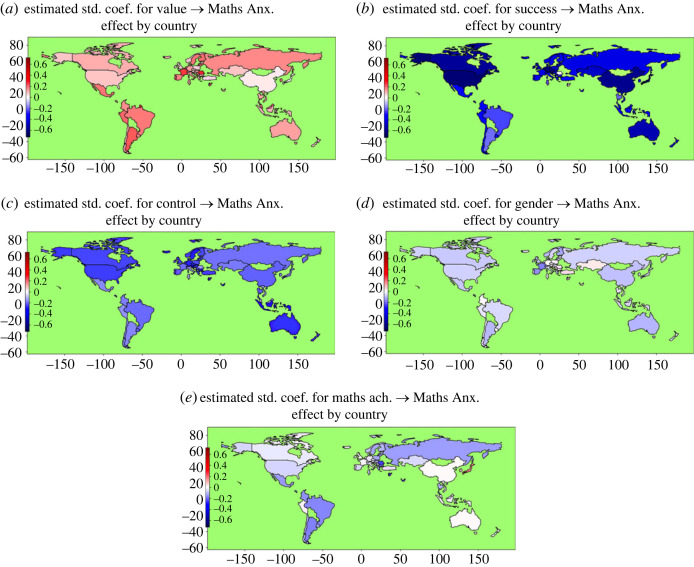
The estimated main effect of each predictor of interest on MA across countries.

Figure 5 illustrates the estimated effect of each predictor of interest on MA across countries. The coefficients are shown numerically in electronic supplementary material, table S1. Success Expectancy (range: 0.066 to −0.693) and Control (range: −0.127 to −0.441) coefficients were negative in nearly all countries in our sample. Their range indicates considerable country-wise variation, however. Positive coefficients for Value (range: 0.022 to 0.292) were especially high (greater than or equal to 0.2) in Argentina, Romania, France, Luxembourg, Portugal, Finland and The Netherlands among other countries. Coefficients were relatively low (less than or equal to 0.1) in Japan, China, Ireland, Norway, the UK, Singapore, Turkey and the USA. Maths Achievement coefficients were mostly negative (in 87% of countries), but with a large range (−0.256, 0.173); notable exceptions were found in East Asian countries, with positive coefficients greater than 0.1 in Japan and South Korea. It should be noted, however, that these coefficients are net of (i.e. controlled for) all other predictors in the model, while the bivariate correlations between MA and Maths Achievement were still negative in every single country, including Japan and South Korea.

In our model, Gender coefficients were negative in most countries, with some exceptions including Jordan, Serbia, Croatia, Romania, Austria, Montenegro and Turkey. The positive coefficients (0 ≤ × ≤ 0.075) in these countries indicate stronger MA in boys than girls. The negative gender coefficients in the other countries indicate stronger MA in girls than boys. The most negative (−0.20 < × < −0.10) coefficients were found in Costa Rica, Chinese Taipei, France, Spain, Belgium, Argentina, Australia, Luxembourg, Switzerland, and Finland. Coefficients were closest to zero in countries such as Germany, Israel and The Netherlands.

Electronic supplementary material, figure S2 shows the mean variable levels in each country (these results are consistent with previously published ones [21]): MA was higher in Latin America, Southern Europe, France and some East Asian countries including Japan than in other countries. Consistent with this, Value was relatively high in most of the high MA countries (except in France and Japan) while Success Expectancy and Control was relatively low in these countries.

## 4. Discussion

Previous studies suggested that subjective factors are probably key in the genesis of MA [24]. Using data from 151 745 fifteen-year-old children from the PISA 2012 database [21], we have determined how subjectively perceived Control over maths activities, the Value of maths and Success Expectancy in maths relate to MA. Our theory-driven investigation is based on EVT and CVT that provide complementary frameworks for the study of MA by highlighting its above three potential predictor variables. We examined main effects, interactions, potential nonlinearities and also considered gender effects. We explored cultural variation in the data from 65 observation units.

In our initial modelling attempt we separated the perceived self-focused (Interest in maths and Utility value) and socially focused (the perceived valuation of maths by parents and friends) valuations of maths [35]. However, this model did not offer a very good fit to the data and the self-focused and socially focused Value constructs were virtually indistinguishable at the latent variable level. A simpler model with a single Value construct (taking into account Interest in maths, Utility of maths, Parents' and Friends’ valuations of maths) offered a good fit to the data. Hence, our final model confirmed the construct choices of EVT and CVT and relied on Control, Value and Success Expectancy factors. We found that interaction effects between predictor variables were practically negligible, main effects well explained the data.

All subjective factors (Success Expectancy, Control and Value perceptions) had notably stronger model coefficients with MA than maths achievement (−0.42, −0.24, + 0.14, versus −0.08). This suggests that subjective self-perceptions indeed have a notably stronger link to MA than ‘objective’ maths achievement. Replicating findings from previous, smaller, studies we found that the higher was Control [34,43] and Success Expectancy [10,33–35,43–48,55,56], the lower was MA. Notably, the original formulation of CVT assumed that ’medium’ level of control was associated with high anxiety ([25], table 1). This is a reasonable expectation as medium levels of control would probably reflect feelings of uncertainty that may lead to anxiety [57]. However, the above empirical studies have indicated a linear relationship between Control and MA. Here, we have examined potential nonlinearities in the MA versus predictor variable relationships and found them to be very modest. Most importantly, the lowest, rather than medium level of Control was associated with the highest-level MA. Success Expectancy had the strongest coefficient effect size, only slightly smaller than its zero-order correlation with MA.

Regarding Value, we found that it had weak negative zero-order correlation with MA (*r* = −0.16). However, multi-level regression modelling found that regression coefficients between Value and MA were weakly positive (*β* = 0*.*14). This pattern of findings is in line with two previous large studies that also reported similar effect sizes to those found here (*r* = −0*.*16 and *r* = −0*.*06; path coefficients = 0.20 and 0.34) in 1219 Portuguese 11- to 16-year-olds and 2648 Singaporean 14-year-olds [46,47]. A third, smaller, study also reported a similar pattern (*r* = −0*.*52; path coefficient = 0.33) in 116 Finnish 13- and 14-year-olds [10].

We suggest that zero-order correlations and regression coefficients are discrepant because Value itself is strongly positively correlated with Success Expectancy and mildly positively correlated with Control. Hence, the positive MA versus Value zero-order correlation is driven by the Value versus Success Expectancy and Control correlations. In contrast to zero-order correlations, regression main effect coefficients represent the change in the outcome variable in response to a unit change in a predictor when other predictors are constant. That is, when the levels of Success Expectancy and Control are fixed, then Value has a positive relationship with MA. Our findings clarify the subjective context of MA: on the average, students with high Success Expectancy also have high Control over maths, tend to attribute higher Value to maths and have less MA than students with lower Success Expectancy and Control. However, if two students have similar levels of Success Expectancy and Control then the one with higher Value will have higher MA than the other one. Higher Value could lead to higher MA because students who value maths more than others probably also perceive maths tests as higher stakes, and therefore more anxiety inducing, events than others.

The coefficient pattern we found also implies that students with the highest level of Value and simultaneously with the lowest level of Control and Success Expectancy would have the highest level of MA. This finding is consistent with the suggestion that MA and other academic anxieties appear when children value a subject highly or attribute high importance to performing well in a subject but at the same time they have *relatively* low Success Expectancy or they rate their ability *relatively* low in a subject [10,32,35].

While our findings are in excellent agreement with the above-cited studies, others reported discrepant outcomes. Lauermann *et al*. [35] found positive correlations and path coefficients between different types of Value measures and worries about mathematics and reading during the actual school year in 8- to 11-year-old US children (0*.*11 *≤* *r* *≤* 0*.*24; *≤* 0*.*04 path coefficients *≤* 0*.*24). Meece *et al*. [44] found negative zero-order correlations between items measuring Value (importance) and MA (−0.12 *≤* *r* *≤* −0.3) and also a negative path coefficient (−0.32) between Value and MA in 250 Grade 7 to 9 US children. The authors noted that evidence was insufficient to explain this relationship. A 1-year longitudinal study reported negative correlations between T1 and T2 Value and MA (*r* = *−*0*.*35 and *r* = *−*0*.*39) and the longitudinal path coefficient between T1 MA and T2 Value was also negative (−0.08) but statistically non-significant in 1242 Year 5 English children [34]. The authors argued that the negative path coefficient may be an artefact due to the correlation of Control and Value. Or, they raised the possibility that if students worry about maths and their Success Expectancy is low then they may consequently devalue maths in order to protect their overall self-esteem [34]. This would also explain the strong negative correlations: students with high MA would attribute low Value to maths. Obviously, measurement-method, age, sample and country-wise variations can contribute to varying findings in studies. We address these issues below.

### 4.1. Cultural effects

Earlier studies from Portugal [46], Finland [10] and Singapore [47] reported positive coefficients for Value whereas a UK [34] and a US study [44] reported negative path coefficients. Another US study reported positive path coefficient [35]. It is interesting to speculate that country-level weak positive Value coefficients in our data may indicate more sample-by-sample variability within some countries (e.g. in the USA and the UK) than other ones: consequently, the signs of the path coefficients reported from these countries may be highly variable, averaging the country-level coefficient closer to zero. One reason for large within-country variation may be that samples from some countries may be more discrepant from each other in attitudes towards maths, e.g. in some countries and some samples devaluing maths may be more acceptable than in others, constituting a mechanism leading to negative Value path coefficients [34].

A notable feature of the PISA 2012 data is that students in China, Japan and Singapore demonstrate very high maths achievement when compared with students from other countries, but they also have much higher MA than expected based on their achievement level compared with the mainstream of countries ([21], p. 102). With regard to this it is important to see that one's high achievement does not necessarily mean that their *subjective* Success Expectancy is also very high. For example, children in many countries have similar self-efficacy perceptions to US children ([21], p. 90) whereas on the PISA test they performed much better ([21], p. 102; fig. III.4.14) than US children (e.g. Chinese Taipei; Hong-Kong, China; Macao-China; Poland). On the one hand, self-efficacy perceptions depend on measurement. However, ceiling effects are unlikely to have happened in the data as students in Shanghai and Singapore did report much higher self-efficacy than US children [21]. So, it is important to consider subjective factors: self-efficacy and self-concept perceptions depend on the subjective definition of success that is related to individual and environmental ’standards of excellence’ defining achievement levels [26]: even if some students perform superbly according to international standards of excellence, their ’home standards of excellence’ and the perceived stakes may be much higher than international standards. For example, Chinese students face extremely tough competition from equally capable fellow students, relentless parental pressure to achieve the highest performance levels (e.g. getting 99% of solutions correct may be considered inadequate) and extremely stringent and selective entrance tests to universities securing the best future career options. These subjective factors probably explain the relatively high MA of Chinese students.

In certain samples other mechanisms may also contribute to dissociating achievement and self-perceptions. For example, UK interview studies found that MA may appear when a well-performing student is moved into a top-level study group. In such cases students may perceive themselves as less able than the already established members of their new reference group [24]. Hence, ’objectively’ high maths performance does not guarantee high maths self-concept. This probably also contributes to gender difference in MA as discussed below.

### 4.2. Gender

Gender was not the focus of our investigation but many studies reported higher MA in girls than boys [18,20,22] and the PISA 2012 data had the same pattern [21], so gender must be considered as an important variable. As reported earlier, MA was higher in girls than in boys in nearly 90% of countries [21]. However, our modelling showed that gender effects were notably discrepant considering countries at the extreme ends of the distribution. Overall, gender effects on MA remained very mild, gender coefficients had notably smaller effect size than Control, Value and Success Expectancy coefficients.

Often finding higher MA in females than males has been a stubbornly stable result during the past half century [16,58]. The gender discrepancy is unlikely to be rooted in achievement difference as MA is higher in girls, even when they perform at the same level as boys [18,20,22]. Several potential, not mutually exclusive, causes could contribute to the gender discrepancy: in line with the CVT and EVT framework used here, the PISA 2012 dataset indicates lesser maths self-concept in girls than boys [21] and girls may experience less pride and enjoyment in maths than boys but more anxiety, hopelessness and shame [32]. So, lesser subjective perception of Control and Success Expectancy could result in increased MA in girls in comparison with boys. While the frequent stereotype that maths is a male domain may contribute to girls' lesser confidence in maths, girls actually tend to report higher MA in more than in less gender-equal countries. In addition, maths gender stereotypes are stronger in many more gender-equal countries than in less gender-equal ones [59]. This ‘gender equality paradox’ suggests that simply increasing gender parity will not lead to decreasing MA, in line with the fact that the gender gap in MA remained stable in Western countries during the past 50 years, contrary to increasing gender equality in these countries [16].

In addition to the above, girls may also be more susceptible to develop higher anxiety in general than boys (be it for social or biological reasons) as studies showed higher general anxiety for everyday events in girls than in boys [18,23]. Girls may also have better meta-cognitive readiness to recognize their own anxiety and they may also be more willing to report their anxiety than boys in self-report questionnaires [22]. Notably, girls may not report higher state MA (measured right before tests) than boys even if they show higher trait MA [50,60]. This discrepancy may be explained by the lower maths self-concept in girls that may affect their long-term self-perceptions about their trait MA but not their state MA [50].

### 4.3. Intervention

Our results suggest that interventions aiming to improve students' maths skills may be beneficial to reducing MA provided that these interventions are designed to improve students’ self-efficacy, self-concept and control perceptions [61–64]. Such improvements will probably come if students' understanding of concepts and why they do what they do in maths improves and they are able to understand the usefulness of maths for everyday problems [7,62]. It has been suggested that frequent formative assessment (given during the learning process with the intention of giving students useful feedback on which they can act) as opposed to summative assessment (given at the end of the learning process usually in the form of a high-stakes, graded exam) is an efficient means to improve Success Expectancy and Control [62]. In some cases, e.g. with high performing students whose maths self-concept still stays relatively low (often in girls), learning about emotion regulation strategies should be beneficial [63]. Notably, different kinds of MA intervention strategies probably suit different groups best [7].

Our results also suggest, seemingly paradoxically, that interventions solely focusing on enhancing the Value of maths would be counterproductive: emphasizing the Value of maths without increasing Control and Success Expectancy is only likely to induce more MA by (explicitly or implicitly) emphasizing the high stakes of maths exams without providing the necessary basis to cope with such exams. Hence, educators should not overemphasize the importance of mathematics to start with. Rather, the positive correlation of Value and the other constructs suggest that the subjective Value of maths can be built up ‘naturally’ by students themselves as their self-efficacy, self-concept and Control perceptions improve.

### 4.4. Limitations and future research directions

Future studies could further refine the measurements of the constructs focused on here and their antecedents. First, Control perceptions, self-efficacy and self-concept in a domain depend on mastery experience, frames of reference, causal control attributions, appraisals from others, physiological reactions in a situation, autonomy expectations, goal setting by parents and others [13,25,26,30,40,41]. Studies could consider these factors as antecedents of Success Expectancy and Control perceptions.

Second, the subjective Value of maths partially depends on the perceived subjective benefits associated with it [13]. The measures used here focused on these perceived benefits: students may value maths positively because they are intrinsically interested in it and enjoy maths activities; they believe it has utility value (e.g. it provides financial or career benefits); or they judge that significant others would appreciate if they do well in it (e.g. because their parents expect this or most of their friends appreciate it). For example, parental valuing of subjects correlates with children's subject valuing and is indirectly linked to children's performance-related worries about mathematics [35]. Future studies could aim to further separate these various contributions to maths valuations and they could also aim to understand how parents', teachers' and friends’ valuations shape the intrinsic and utility Value of maths on the long run.

Third, the Value of maths is not only shaped by perceived benefits but also by the perceived costs associated with maths [13,65,66]. Students may find maths too costly and consequently value it negatively if it is thought to have too high ’opportunity cost’, restricting alternative choices too much (e.g. students cannot do other preferred school or extracurricular activities while they study maths); if they find maths difficult (requiring too much mental effort); or, if it is associated with too much anxiety (emotional cost). The measurement of the perceived costs of maths has been neglected so far and they were also not measured in the current data [65].

Fourth, subjective values are unlikely to be based solely on rational cost/benefit analyses. The above-mentioned emotional costs may be hard to control or justify rationally. Emotional factors are also often based on personal preferences not directly linked to a subject's content, for example, a subject may be valued because of the liking of a teacher [67]. Or, it may not be possible to rationalize internalized values and maths anxious attitudes learnt from parents [68,69]. Genetic factors and temperament may also influence achievement emotions [25,26,30] and may make some students more susceptible to develop academic anxieties than others [23]. Future studies could explore the relevant personal emotional and biological factors.

Fifth, as the above discussion implies, Control, Success Expectancy and Value are not exclusively causal factors; they can also be affected by MA and by emotions in general. Such multi-directional and potentially complex relationships are difficult to understand, similar to the question of whether MA is rooted in poor performance, or, whether MA leads to poor performance [31]. Disentangling complicated causal relations requires longitudinal studies of which very few are available [34].

Sixth, while the perception of anxiety is probably subject to cultural variation, MA is mostly measured by self-report questionnaires that can be affected by different biases in different cultures. However, we know very little about cultural variation in the perception and reporting of MA. Further cross-cultural comparisons can help us understand these.

Finally, the PISA 2012 dataset includes data from 15-year-olds collected more than 10 years ago in 2012. More recent relevant data from other age groups should also be examined.

### 4.5. Conclusions

Taking EVT and CVT as theoretical background, focusing on subjective measures of Control, Value and Success Expectancy in maths provides a promising framework for understanding subjective factors probably key to the genesis of MA. A more nuanced clarification of the antecedents of these constructs and how relevant factors interact and develop in various populations and age groups can lead us closer to developing effective methods for alleviating or preventing MA.

## Data Availability

The data can be accessed at: https://www.oecd.org/pisa/data/pisa2012database-downloadabledata.htm. Supplementary material is available online [70].

## References

[1] Jalongo MR, Hirsh RA. 2010 Understanding reading anxiety: new insights from neuroscience. Early Childhood Edu. J. **37**, 431-435. (10.1007/s10643-010-0381-5)

[2] Yerkes RM, Dodson JD. 1908 The relation of strength of stimulus to rapidity of habit-formation. J. Comp. Neurol. Psychol. **18**, 459-482. (10.1002/cne.920180503)

[3] Eysenck MW, Calvo MG. 1992 Anxiety and performance: the processing efficiency theory. Cogn. Emot. **6**, 409-434. (10.1080/02699939208409696)

[4] Eysenck MW, Derakshan N, Santos R, Calvo MG. 2007 Anxiety and cognitive performance: attentional control theory. Emotion **7**, 336-353. (10.1037/1528-3542.7.2.336) 17516812

[5] Ashcraft MH, Kirk EP. 2001 The relationships among working memory, math anxiety, and performance. J. Exp. Psychol: Gen. **130**, 224-237. (10.1037/0096-3445.130.2.224) 11409101

[6] Ashcraft MH. 2002 Math anxiety: personal, educational, and cognitive consequences. Curr. Dir. Psychol. Sci. **11**, 181-185. (10.1111/1467-8721.00196)

[7] Szűcs D, Mammarella I. 2020 Mathematics anxiety, educational practices series. Brussels, Belgium: UNESCO IBE.

[8] Mammarella IC, Caviola S, Dowker A (eds). 2019 Mathematics anxiety: what is known and what is still to be understood. London, UK: Routledge.

[9] Dowker A, Sarkar A, Looi CY. 2016 Mathematics anxiety: what have we learned in 60 years? Front. Psychol. **7**, 508. (10.3389/fpsyg.2016.00508) 27199789 PMC4842756

[10] Kyttälä M, Björn PM. 2010 Prior mathematics achievement, cognitive appraisals and anxiety as predictors of Finnish students' later mathematics performance and career orientation. Edu. Psychol. **30**, 431-448. (10.1080/01443411003724491)

[11] Ahmed W. 2018 Developmental trajectories of math anxiety during adolescence: associations with STEM career choice. J. Adolesc. **67**, 158-166. (10.1016/j.adolescence.2018.06.010) 29975882

[12] Wigfield A, Eccles JS, Iver DM, Reuman DA, Midgley C. 1991 Transitions during early adolescence: changes in children's domain-specific self-perceptions and general self-esteem across the transition to junior high school. Dev. Psychol. **27**, 552-565. (10.1037/0012-1649.27.4.552)

[13] Wigfield A, Eccles JS. 2000 Expectancy value theory of achievement motivation. Contemp. Educ. Psychol. **25**, 68-81. (10.1006/ceps.1999.1015) 10620382

[14] Eccles JS, Wigfield A. 2002 Motivational beliefs, values, and goals. Annu. Rev. Psychol. **53**, 109-132. (10.1146/annurev.psych.53.100901.135153) 11752481

[15] Pekrun R, Elliot AJ, Maier MA. 2006 Achievement goals and discrete achievement emotions: a theoretical model and prospective test. J. Educ. Psychol. **98**, 583-597. (10.1037/0022-0663.98.3.583)

[16] Hembree R. 1990 The nature, effects, and relief of mathematics anxiety. J. Res. Math. Educ. **21**, 33. (10.2307/749455)

[17] Ma X, Xu J. 2004 The causal ordering of mathematics anxiety and mathematics achievement: a longitudinal panel analysis. J. Adolesc. **27**, 165-179. (10.1016/j.adolescence.2003.11.003) 15023516

[18] Hill F, Mammarella IC, Devine A, Caviola S, Passolunghi MC, Szűcs D. 2016 Maths anxiety in primary and secondary school students: gender differences, developmental changes and anxiety specificity. Learn. Individ. Differ. **48**, 45-53. (10.1016/j.lindif.2016.02.006)

[19] Caviola S, Toffalini E, Giofrè D, Ruiz JM, Szűcs D, Mammarella IC. 2021 Math performance and academic anxiety forms, from sociodemographic to cognitive aspects: a meta- analysis on 906311 participants. Educ. Psychol. Rev. **34**, 363-399. (10.1007/s10648-021-09618-5)

[20] Devine A, Hill F, Carey E, Szűcs D. 2018 Cognitive and emotional math problems largely dissociate: prevalence of developmental dyscalculia and mathematics anxiety. J. Edu. Psychol. **110**, 431-444. (10.1037/edu0000222)

[21] OECD. 2013 PISA 2012 results: ready to learn: students' engagement, drive and self-beliefs (volume III). Brussels, Belgium: OECD Publishing.

[22] Devine A, Fawcett K, Szűcs D, Dowker A. 2012 Gender differences in mathematics anxiety and the relation to mathematics performance while controlling for test anxiety. Behav. Brain Funct. **8**, 33. (10.1186/1744-9081-8-33) 22769743 PMC3414752

[23] Carey E, Devine A, Hill F, Szűcs D. 2017 Differentiating anxiety forms and their role in academic performance from primary to secondary school. PLoS ONE **12**, e0174418. (10.1371/journal.pone.0174418) 28350857 PMC5370099

[24] Carey E, Devine A, Hill F, Dowker A, McLellan R, Szűcs D. 2019 Understanding mathematics anxiety: investigating the experiences of UK primary and secondary school students. Cambridge, UK: Apollo – University of Cambridge Repository.

[25] Pekrun R. 2006 The control-value theory of achievement emotions: assumptions, corollaries, and implications for educational research and practice. Edu. Psychol. Rev. **18**, 315-341. (10.1007/s10648-006-9029-9)

[26] Pekrun R, Frenzel A, Thomas G, Perry PR. 2007 The control-value theory of achievement emotions: an integrative approach to emotions in education. In Emotions in education (eds PA Schutz, R Pekrun), pp. 13-36. New York, NY: Academic Press.

[27] Goetz T, Frenzel AC, Pekrun R, Hall NC. 2006 The domain specificity of academic emotional experiences. J. Exp. Edu. **75**, 5-29. (10.3200/JEXE.75.1.5-29)

[28] Schukajlow S, Rakoczy K, Pekrun R. 2017 Emotions and motivation in mathematics education: theoretical considerations and empirical contributions. ZDM **49**, 307-322. (10.1007/s11858-017-0864-6) PMC9845103 36684477

[29] Lichtenfeld S, Pekrun R, Stupnisky RH, Reiss K, Murayama K. 2012 Measuring students' emotions in the early years: the achievement emotions questionnaire-elementary school (AEQ-ES). Learn. Individ. Diff. **22**, 190-201. (10.1016/j.lindif.2011.04.009)

[30] Reinhard P, Raymond PP. 2014 Control-value theory of achievement emotions. In International handbook of emotions in education (eds R Pekrun, L Linnenbrink-Garcia), pp. 120-141. London, UK: Routledge.

[31] Carey E, Hill F, Devine A, Szűcs D. 2016 The chicken or the egg? The direction of the relationship between mathematics anxiety and mathematics performance. Front. Psychol. **6**. (10.3389/fpsyg.2015.01987) PMC4703847 26779093

[32] Frenzel AC, Pekrun R, Goetz T. 2007 Girls and mathematics—a ‘hopeless’ issue? A control-value approach to gender differences in emotions towards mathematics. Eur. J. Psychol. Edu. **22**, 497-514. (10.1007/BF03173468)

[33] Lazarides R, Raufelder D. 2020 Control-value theory in the context of teaching: does teaching quality moderate relations between academic self-concept and achievement emotions? Br. J. Educ. Psychol. **91**, 127-147. (10.1111/bjep.12352) 32369196

[34] Putwain DW, Wood P. 2022 Anxiety in the mathematics classroom: reciprocal relations with control and value, and relations with subsequent achievement. ZDM – Mathematics Education **55**, 285-298. (10.1007/s11858-022-01390-2)

[35] Lauermann F, Eccles JS, Pekrun R. 2017 Why do children worry about their academic achievement? An expectancy-value perspective on elementary students' worries about their mathematics and reading performance. ZDM **49**, 339-354. (10.1007/s11858-017-0832-1)

[36] Pajares F. 2002 Gender and perceived self-efficacy in self-regulated learning. Theory Pract. **41**, 116-125. (10.1207/s15430421tip4102_8)

[37] Bandura A. 1977 Self-efficacy: toward a unifying theory of behavioral change. Psychol. Rev. **84**, 191-215. (10.1037/0033-295X.84.2.191) 847061

[38] Bandura A. 1977 Self-efficacy: the excercise of control. New York, NY: W.H. Freeman and Company.

[39] Pajares F, Miller MD. 1994 Role of self-efficacy and self-concept beliefs in mathematical problem solving: a path analysis. J. Educ. Psychol. **86**, 193-203. (10.1037/0022-0663.86.2.193)

[40] Bong M, Clark RE. 1999 Comparison between self-concept and self-efficacy in academic motivation research. Educ. Psychol. **34**, 139-153. (10.1207/s15326985ep3403_1)

[41] Bong M, Skaalvik EM. 2003 Academic self-concept and self-efficacy: how different are they really? Educ. Psychol. Rev. **15**, 1-40. (10.1023/A:1021302408382)

[42] Putwain DW, Pekrun R, Nicholson LJ, Symes W, Becker S, Marsh HW. 2018 Control-value appraisals, enjoyment, and boredom in mathematics: a longitudinal latent interaction analysis. Am. Educ. Res. J. **55**, 1339-1368. (10.3102/0002831218786689)

[43] Putwain DW, Schmitz EA, Wood P, Pekrun R. 2021 The role of achievement emotions in primary school mathematics: control–value antecedents and achievement outcomes. Br. J. Educ. Psychol. **91**, 347-367. (10.1111/bjep.12367) 32662521

[44] Meece JL, Wigfield A, Eccles JS. 1990 Predictors of math anxiety and its influence on young adolescents’ course enrollment intentions and performance in mathematics. J. Educ. Psychol. **82**, 60-70. (10.1037/0022-0663.82.1.60)

[45] Ahmed W, Minnaert A, Kuyper H, van der Werf G. 2012 Reciprocal relationships between math self-concept and math anxiety. Learn. Individ. Differ. **22**, 385-389. (10.1016/j.lindif.2011.12.004)

[46] Peixoto F, Sanches C, Mata L, Monteiro V. 2016 ‘How do you feel about math?’: relationships between competence and value appraisals, achievement emotions and academic achievement. Eur. J. Psychol. Educ. **32**, 385-405. (10.1007/s10212-016-0299-4)

[47] Luo W, Ng PT, Lee K, Aye KM. 2016 Self-efficacy, value, and achievement emotions as mediators between parenting practice and homework behavior: a control-value theory perspective. Learn. Individ. Differ. **50**, 275-282. (10.1016/j.lindif.2016.07.017)

[48] Clem A-L, Hirvonen R, Aunola K, Kiuru N. 2021 Reciprocal relations between adolescents' self-concepts of ability and achievement emotions in mathematics and literacy. Contemp. Educat. Psychol. **65**, 101964. (10.1016/j.cedpsych.2021.101964)

[49] OECD. 2018 Education at a glance. Brussels, Belgium: OECD.

[50] Bieg M, Goetz T, Lipnevich AA. 2014 What students think they feel differs from what they really feel – academic self-concept moderates the discrepancy between students’ trait and state emotional self-reports. PLoS ONE **9**, e92563. (10.1371/journal.pone.0092563) 24647760 PMC3960260

[51] R Core Team. 2022 R: A language and environment for statistical computing. Vienna, Austria: R Foundation for Statistical Computing.

[52] Van Rossum G, Drake FL. 2009 *Python 3 reference manual*. Scotts Valley, CA: CreateSpace.

[53] Bafumi J, Gelman A. 2007 Fitting multilevel models when predictors and group effects correlate. SSRN Elect. J. (10.2139/ssrn.1010095)

[54] OECD. 2009 PISA data analysis manual: SPSS, 2nd edn. Brussels, Belgium: OECD.

[55] Jameson MM. 2014 Contextual factors related to math anxiety in second-grade children. J. Exp. Educ. **82**, 518-536. (10.1080/00220973.2013.813367)

[56] Gopal K, Salim N, Ayub A. 2020 Study on mathematics self-efficacy and anxiety among Malaysian upper secondary students using fuzzy conjoint analysis. Malaysian J. Math. Sci. **14**, 63-79.

[57] Grupe DW, Nitschke JB. 2013 Uncertainty and anticipation in anxiety: an integrated neurobiological and psychological perspective. Nat. Rev. Neurosci. **14**, 488-501. (10.1038/nrn3524) 23783199 PMC4276319

[58] Betz NE. 1978 Prevalence, distribution, and correlates of math anxiety in college students. J. Couns. Psychol. **25**, 441-448. (10.1037/0022-0167.25.5.441)

[59] Stoet G, Geary DC. 2018 The gender-equality paradox in science, technology, engineering, and mathematics education. Psychol. Sci. **29**, 581-593. (10.1177/0956797617741719) 29442575

[60] Bieg M, Goetz T, Hubbard K. 2013 Can I master it and does it matter? An intraindividual analysis on control–value antecedents of trait and state academic emotions. Learn. Individ. Differ. **28**, 102-108. (10.1016/j.lindif.2013.09.006)

[61] Elbaum B, Vaughn S. 2001 School-based interventions to enhance the self-concept of students with learning disabilities: a meta-analysis. Elem. Sch. J. **101**, 303-329. (10.1086/499670)

[62] Klee HL, Buehl MM, Miller AD. 2021 Strategies for alleviating students' math anxiety: control-value theory in practice. Theory Pract. **61**, 49-61. (10.1080/00405841.2021.1932157)

[63] Tze V, Parker P, Sukovieff A. 2021 Control-value theory of achievement emotions and its relevance to school psychology. Can. J. Sch. Psychol. **37**, 23-39. (10.1177/08295735211053962)

[64] Passolunghi MC, Vita CD, Pellizzoni S. 2020 Math anxiety and math achievement: the effects of emotional and math strategy training. Dev. Sci. **23**, e12964. (10.1111/desc.12964) 32159906

[65] Hentges RF, Galla BM, Wang M-T. 2018 Economic disadvantage and math achievement: the significance of perceived cost from an evolutionary perspective. Br. J. Educ. Psychol. **89**, 343-358. (10.1111/bjep.12242) 30187455

[66] Eccles JS, Wigfield A. 2020 From expectancy-value theory to situated expectancy-value theory: a developmental, social cognitive, and sociocultural perspective on motivation. Contemp. Educ. Psychol. **61**, 101859. (10.1016/j.cedpsych.2020.101859)

[67] Semeraro C, Giofre D, Coppola G, Lucangeli D, Cassibba R. 2020 The role of cognitive and non-cognitive factors in mathematics achievement: the importance of the quality of the student-teacher relationship in middle school. PLoS ONE **15**, e0231381. (10.1371/journal.pone.0231381) 32310988 PMC7170247

[68] Maloney EA, Ramirez G, Gunderson EA, Levine SC, Beilock SL. 2015 Intergenerational effects of parents’ math anxiety on children's math achievement and anxiety. Psychol. Sci. **26**, 1480-1488. (10.1177/0956797615592630) 26253552

[69] Szczygiewl M. 2020 When does math anxiety in parents and teachers predict math anxiety and math achievement in elementary school children? The role of gender and grade year. Soc. Psychol. Educ. **23**, 1023-1054. (10.1007/s11218-020-09570-2)

[70] Szucs D, Toffalini E. 2023 Maths anxiety and subjective perception of control, value and success expectancy in mathematics. *Figshare*. (10.6084/m9.figshare.c.6935833) PMC10685112 38034120

